# Factor Tree Copula Models for Item Response Data

**DOI:** 10.1007/s11336-023-09917-6

**Published:** 2023-06-01

**Authors:** Sayed H. Kadhem, Aristidis K. Nikoloulopoulos

**Affiliations:** School of Computing Sciences, University of East Anglia, Norwich, NR4 7TJ UK

**Keywords:** conditional dependence, factor copula models, latent variable models, truncated vine copula models

## Abstract

Factor copula models for item response data are more interpretable and fit better than (truncated) vine copula models when dependence can be explained through latent variables, but are not robust to violations of conditional independence. To circumvent these issues, truncated vines and factor copula models for item response data are joined to define a combined model, the so-called factor tree copula model, with individual benefits from each of the two approaches. Rather than adding factors and causing computational problems and difficulties in interpretation and identification, a truncated vine structure is assumed on the residuals conditional on one or two latent variables. This structure can be better explained as a conditional dependence given a few interpretable latent variables. On the one hand, the parsimonious feature of factor models remains intact and any residual dependencies are being taken into account on the other. We discuss estimation along with model selection. In particular, we propose model selection algorithms to choose a plausible factor tree copula model to capture the (residual) dependencies among the item responses. Our general methodology is demonstrated with an extensive simulation study and illustrated by analyzing Post-Traumatic Stress Disorder.

Factor or conditional independence models are widely called for analyzing item response data using much fewer unobserved/latent variables or factors (Bartholomew et al., 2011). These are natural if the dependence amongst the *d* observed variables or items is assumed to arise from *p* latent variables with $p<<d$. They are parsimonious models and favorable for large dimensions as the number of parameters is $\mathcal {O}(d)$ instead of $\mathcal {O}(d^2)$, as for, e.g., in discretized multivariate normal (MVN) models with unstructured correlation matrices (e.g., Muthén 1978; Maydeu-Olivares 2006). Nevertheless, factor models mainly assume that the items are conditionally independent given some latent variables. This assumption implies that the dependence amongst the observed variables is fully accounted for by the factors with no remaining dependence. This could lead to biased estimates if the strict assumption of conditional independence is violated (Braeken et al., 2007; Sireci et al., 1991; Chen and Thissen, 1997; Yen, 1993). The conditional independence assumption is violated if there exists local or residual dependence. Mitigating the residual dependence might be achieved by adding more latent variables to the factor model, but at the expense of computational problems and difficulties in interpretation and identification.

To circumvent these problems, the items can be allowed to interrelate by forming a dependence structure with conditional dependence given a few interpretable latent variables. In this way, on the one hand the parsimonious feature of factor models remains intact and any residual dependencies are being taken into account on the other. This can be achieved by incorporating copulas into the conditional distribution of factor models in order to provide a conditional dependence structure given very few latent variables. Such copula approaches for item response data are proposed by Braeken et al. (2007, 2013) and Braeken (2011) who explored the use of Archimedean copulas or a mixture of the independence and comonotonicity copulas to capture the residual dependence of traditional item response theory models. Therein simple copulas have been used for subgroups of items that are chosen from the context with homogeneous within-subgroup dependence. This is due to the fact that Archimedean copulas allow only for exchangeable dependence with a narrower range as the dimension increases (McNeil and Neslehova, 2009).

Without a priori knowledge of obvious subgroups of items that are approximately exchangeable, we will propose a more general residual dependence approach that makes the use of truncated regular vine copula models (Brechmann et al., 2012) to construct the conditional distribution of factor models. Within a vine copula specification, no such restrictions need to be made. To define the conditional independence part of the model, we also use truncated vine copulas rather than the traditional factor models for item response in Braeken et al. (2007, 2013) and Braeken (2011). Nikoloulopoulos and Joe (2015) have proposed factor copula models for item response data. These factor models can be explained as truncated C-vines rooted at the latent variables. The C-vine is a boundary case of regular vine copulas, which is suitable if there exists a (latent) variable that drives the dependence among the items (Nikoloulopoulos et al., 2012). For the first factor, there are bivariate copulas that couple each item to the first latent variable and for the second factor there are copulas that link each item to the second latent variable conditioned on the first factor (leading to conditional dependence parameters), etc. Factor copula models with appropriately chosen linking copulas will be useful when the items (a) have more probability in joint upper or lower tail than would be expected with a discretized MVN, or (b) can be considered as discretized maxima/minima or mixtures of discretized means rather than discretized means. For different bivariate copulas, the middle part of the item characteristic curve (ICC) is similar, but can differ more for extreme values of the latent variable because of the different tail behavior of the bivariate copulas (Nikoloulopoulos and Joe, 2015).

The proposed parsimonious approach, that requires no priori knowledge of the subgroups of items, can be explained as a truncated regular vine copula model that involves both observed and latent variables, but, more simply, we derive the models as conditional dependence models with a few interpretable latent variables that model the residual dependence of the factor copula model via an 1-truncated vine copula. The factor copula model explains most of the dependence and the remaining dependence is further accounted for by an 1-truncated vine copula conditioned on the factors. Brechmann and Joe (2014) and Joe (2018) initiated the study of such conditional dependence models with a unidimensional factor/latent variable for continuous data. The combined 1-factor and 1-truncated vine model for continuous data in Brechmann and Joe (2014) is restricted to Gaussian dependence, but Joe (2018) proposed a combination of an 1-factor copula model with 1-truncated vine copula model with non-Gaussian bivariate copulas. Our models for item response are discrete counterparts of the models in Brechmann and Joe (2014) and Joe (2018) with interpretation (the items can be considered as discretized maxima/minima or mixtures of discretized means rather than discretized means) and technical details that are quite different and provide an extension to more than one factors. Furthermore, we propose heuristic algorithms that automatically select the bivariate parametric copula families and 1-truncated vine tree structure for the proposed 1- and 2-factor tree copula models for item response data.

The remainder of the paper proceeds as follows. In Sect. 1, we introduce the combined factor/truncated vine copula models for item response data. Section 2 provides estimation techniques and computational details. Section 3 discusses 1-truncated vine tree structure and bivariate copula selection. Section 4 has an extensive simulation study to assess the estimation techniques and model selection algorithms. Our methodology is illustrated using real data in Sect. 5. We conclude with some discussion in Sect. 6, followed by a brief section with software details.

## Factor Tree Copula Models for Item Response

This section introduces the theory of the combined factor/truncated vine copula models for item response data. Before that, the first two subsections provide some background about vine and factor copula models for discrete responses.

### Overview and Relevant Background for Copulas

A copula is a multivariate cumulative distribution function (cdf) with uniform *U*(0, 1) margins. If *F* is a *d*-variate cdf with univariate margins $F_1,\ldots ,F_d$, then Sklar’s (1959) theorem implies that there is a copula *C* such that

$$\begin{aligned} F(y_1,\ldots ,y_d)= C\Bigl (F_1(y_1),\ldots ,F_d(y_d)\Bigr ). \end{aligned}$$

The copula is unique if $F_1,\ldots ,F_d$ are continuous, but not if some of the $F_j$ have discrete components. Nevertheless, if $C(\cdot ;\theta )$ is a parametric family of copulas and $F_j(\cdot ;\eta _j)$ is a parametric model with discrete components for the *j*th univariate margin, then

$$\begin{aligned} C\Bigl (F_1(y_1;\eta _1),\ldots ,F_d(y_d;\eta _d);\theta \Bigr ) \end{aligned}$$

is a valid multivariate parametric model with univariate margins $F_1,\ldots ,F_d$.

**Fig. 1:**
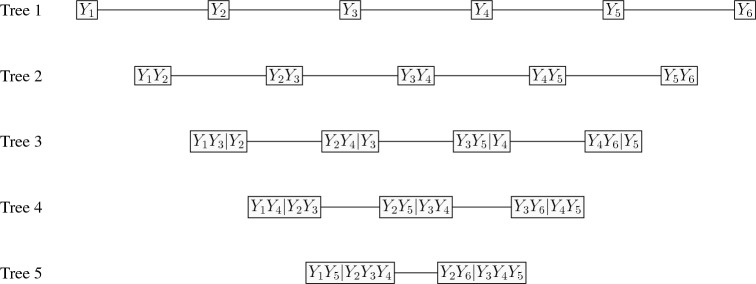
Graphical representation of a D-vine copula model with 6 variables and 5 trees.

The choice of the parametric family of copulas could not be other than the class of regular vine copulas (Bedford and Cooke, 2002) as other parametric copulas such as Archimedean, nested Archimedean and elliptical copulas have limited dependence (Nikoloulopoulos, 2013). Regular vine copulas are a flexible class of models that are constructed from a set of bivariate copulas in hierarchies or tree levels (Joe, 1996; Bedford and Cooke, 2001, 2002; Kurowicka and Cooke, 2006; Kurowicka and Joe, 2011; Joe, 2014; Gronneberg and Foldnes, 2017; Gronneberg et al., 2022). The *d*-dimensional regular vine copulas are built via successive mixing from $d(d -1)/2$ bivariate linking copulas on trees. They involve $d-1$ trees, the first tree represents dependence (as edges) amongst *d* variables (as nodes). Then, the edges become nodes in the next tree, involving the conditional dependencies given a common variable. This process continues until tree $d - 1$ that includes two nodes and one edge, representing conditional dependence of two variables given $d - 2$ variables (Chang and Joe, 2019). A *d*-dimensional regular vine copula can cover flexible dependence structures, different from assuming simple linear correlation structures, tail independence and normality (Nikoloulopoulos et al., 2012), through the specification of $d-1$ bivariate parametric copulas at tree 1 and $$\left( {\begin{array}{c}d-1\\ 2\end{array}}\right) $$ bivariate conditional parametric copulas at higher trees; at tree $\ell$ for $\ell =2,\ldots ,d-1$, there are $d-\ell$ bivariate conditional copulas that condition on $\ell -1$ variables. Depending on the types of trees, various regular vine copulas can be constructed. Two boundary cases are D-vines and C-vines. In Fig. 1, a D-vine with 6 variables and 5 trees is depicted, where the bivariate pairs at tree 1 are $Y_j,Y_{j+1}$, for $j=1,\ldots ,5$, and for tree $\ell$ ($2\le \ell <6$), the (conditional) bivariate pairs are $Y_j,Y_{j+\ell }|Y_{j+1},\ldots ,Y_{j+\ell -1}$ for $j=1,\ldots ,6-\ell$. That is, for the D-vine, conditional bivariate copulas are specified for variables *j* and $j+\ell$ given the variables indexed in between.

Joe et al. (2010) have shown that in order for a vine copula to have (tail) dependence for all bivariate margins, it is only necessary for the bivariate copulas in tree 1 to have (tail) dependence and it is not necessary for the conditional bivariate copulas in trees $2,\ldots ,d-1$ to have (tail) dependence. That provides the theoretical justification for the idea to model the dependence in the first trees and then, just use the independence copulas to model conditional dependence at higher trees without sacrificing the tail dependence of the vine copula distribution. This truncation, as per the terminology in Brechmann et al. (2012), provides a parsimonious vine copula model. The $\ell$-truncated vine copula (truncated after tree $\ell$) can provide, with appropriately chosen linking copulas, asymmetric dependence structure as well as tail dependence (dependence among extreme values). Joe et al. (2010) have shown that by choosing bivariate linking copulas appropriately, vine copulas can have a flexible range of lower/upper tail dependence and different lower/upper tail dependence parameters for each bivariate margin.

In the context of multivariate discrete data, upper or lower tail dependence means that more probabilities can be assigned in the joint upper or joint lower tails. Hence, choices of copulas with upper or lower tail dependence are better if the items have more joint upper or lower tail probability than would be expected with the discretized MVN model (Muthén, 1978). Note in passing that the discretized MVN distribution is a special case of the vine copula model with discrete margins. If all bivariate copulas are bivariate normal (BVN) in the vine copula model, then the resulting model is the discretized MVN.

### Factor Copula Models

Let $\textbf{Y}=\{Y_{1}, \ldots ,Y_{d}\}$ denote the vector with the item response variables that are all measured on an ordinal scale; $Y_{j}\in \{0,\ldots ,K_{j}-1\}$. Let the cutpoints in the uniform *U*(0, 1) scale for the *j*th item be $a_{j,k}$, $k=1,\ldots ,K-1$, with $a_{j,0}=0$ and $a_{j,K}=1$. These correspond to $a_{j,k}=\Phi (\alpha _{j,k})$, where $\alpha _{j,k}$ are cutpoints in the normal *N*(0, 1) scale.

The *p*-factor model assumes that $\textbf{Y}$, with corresponding realizations $\textbf{y}=\{y_{1}, \ldots ,y_{d}\}$, is conditionally independent given the *p*-dimensional latent vector $\textbf{X}=(X_1,\ldots ,X_p)$. The joint probability mass function (pmf) of the *p*-factor model is

1 $$\begin{aligned} \pi _d(\textbf{y})=\Pr (Y_1=y_1,\ldots ,Y_d=y_d)= \int \prod _{j=1}^d\Pr (Y_j=y_j|X_1=x_1, \ldots ,X_p=x_p)\, \hbox {d}F_{\textbf{X}}(x), \end{aligned}$$

where $F_{\textbf{X}}$ is the distribution of the latent vector $\textbf{X}$. The factor copula methodology (Nikoloulopoulos and Joe, 2015) uses a set of bivariate copulas that link the items to the latent variables to specify $\Pr (Y_j=y_j|X_1=x_1, \ldots ,X_p=x_p)$. Below we include the theory for one and two factors.

For the 1-factor model, let $X_1$ be a latent variable that is standard uniform. From Sklar (1959), there is a bivariate copula $C_{X_1j}$ such that $\Pr (X_1\le x, Y_j\le y)=C_{X_1j}\bigl (x,F_j(y)\bigr )$ for $0\le x\le 1$ where $F_j(y)=a_{j,y+1}$ is the cdf of $Y_j$. Then, it follows that

2 $$\begin{aligned} F_{j|X_1}(y|x):=\Pr (Y_j\le y|X_1=x) = {\partial C_{X_1j}(x,a_{j,y+1})\over \partial x}=C_{j|X_1}(a_{j,y+1}|x). \end{aligned}$$

Hence, the pmf for the 1-factor copula model becomes

$$\begin{aligned} \pi _d(\textbf{y})= & {} \int _0^1\prod _{j=1}^d\Pr (Y_j=y_j|X_1=x)\,\hbox {d}x =\int _0^1\prod _{j=1}^d f_{j|X_1}(y_j|x) \,\hbox {d}x, \end{aligned}$$

where

3 $$\begin{aligned} f_{j|X_1}(y|x)= C_{j|X_1}(a_{j,y+1}|x) - C_{j|X_1}(a_{j,y}|x). \end{aligned}$$

Note in passing that (3) is the ICC for the 1-factor copula model. The copula $C_{X_1j}$ controls the shape of the ICC.

**Fig. 2:**
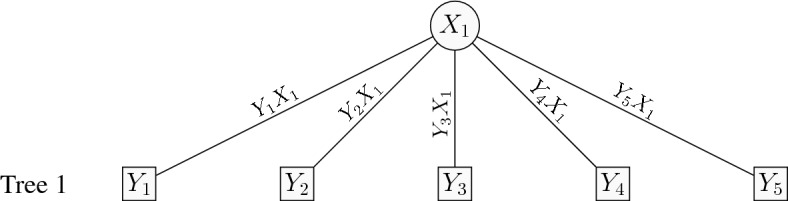
Graphical representation of an 1-factor copula model with $d=5$ items.

The 1-factor copula model can be explained as an 1-truncated C-vine copula model rooted at the latent variable $X_1$. For the *d*-dimensional 1-factor copula model, the pairs at tree 1 are $Y_jX_1$ for $j=1,\ldots ,d$ and for higher trees the (conditional) copula pairs are set to independence. That is the 1-factor copula model has *d* bivariate copulas $C_{X_1j}$ that link $Y_j,\,j=1,\ldots ,d$ with $X_1$ in the 1st tree of the C-vine, and independence copulas in all the remaining trees of the C-vine (truncated after the 1st tree). From the results in Joe et al. (2010) and Krupskii and Joe (2013), upper or lower tail dependent copulas in tree 1 will lead to items that have more probability in joint upper or lower tail than would be expected with a discretized MVN. Figure 2 depicts the graphical representation of a 1-factor copula model with $d=5$ items as an 1-truncated C-vine.

For the 2-factor copula model, let $X_1,X_2$ be latent variables that are independent uniform *U*(0, 1) random variables. Let $C_{X_1j}$ be defined as in the 1-factor copula model and $C_{X_2j}$ be a bivariate copula such that

$$\begin{aligned} \Pr (X_2\le x_2,Y_j\le y|X_1=x_1) =C_{X_2j}\bigl (x_2,F_{j|X_1}(y|x_1)\bigr ), \end{aligned}$$

where $F_{j|X_1}$ is given in (2). Here, we are making the simplifying assumption that the conditional copula for the univariate distributions $F_{X_2|X_1}=F_{X_2}$ and $F_{j|X_1}$ does not depend on $x_1$; this is a model assumption as by Sklar’s theorem there exist such bivariate copulas that in general depend on $x_1\in [0,1]$. Then for $0\le x_1,x_2\le 1$,

4 $$\begin{aligned} F_{X_2j|X_1}(x_2,y|x_1):=\Pr (Y_j&\le y|X_1=x_1,X_2= x_2) = {\partial \over \partial x_2} \Pr (X_2\le x_2,Y_j\le y|X_1=x_1) \nonumber \\&={\partial \over \partial x_2} C_{X_2j}\Bigl (x_2,F_{j|X_1}(y|x_1)\Bigr )= C_{j|X_2}\Bigl (F_{j|X_1}(y|x_1)|x_2\Bigr ). \end{aligned}$$

Hence, the pmf for the 2-factor copula model is

$$\begin{aligned} \pi _d(\textbf{y})= & {} \int _0^1\int _0^1\prod _{j=1}^d\Pr (Y_j=y_j|X_1=x_1,X_2=x_2)\,\hbox {d}x_1 \hbox {d}x_2 \nonumber \\= & {} \int _0^1\int _0^1\prod _{j=1}^d f_{X_2j|X_1}\bigl (x_2,y_j|x_1\bigr )\,\hbox {d}x_1 \hbox {d}x_2, \end{aligned}$$

where

5 $$\begin{aligned} f_{X_2j|X_1}(x_2,y|x_1)= C_{j|X_2}\Bigl (F_{j|X_1}(y|x_1)|x_2\Bigr )-C_{j|X_2}\Bigl (F_{j|X_1}(y-1|x_1)|x_2\Bigr ). \end{aligned}$$

Note in passing that (5) is the ICC for the 2-factor copula model. The copulas $C_{X_1j},C_{X_2j}$ control the shape of the ICC.

**Fig. 3:**
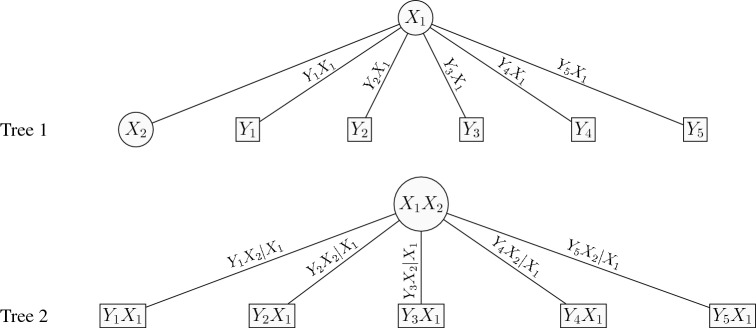
Graphical representation of a 2-factor copula model with $d=5$ items. Note that the factors are linked to one another with an independent copula in Tree 1.

The 2-factor copula model can be explained as a 2-truncated C-vine. For the *d*-dimensional 2-factor copula model, the pairs at tree 1 are $Y_jX_1$ for $j=1,\ldots ,d$, the pairs at tree 2 are $Y_jX_2|X_1$ for $j=1,\ldots ,d$, and for higher trees the (conditional) copula pairs are set to independence. That is the 2-factor copula model has *d* bivariate copulas $C_{X_1j}$ that link $Y_j,\,j=1,\ldots ,d$ with $X_1$ in the first tree of the C-vine, *d* bivariate copulas $C_{X_2j}$ that link $Y_j,\,j=1,\ldots ,d$ with $X_2$ given $X_1$ in the second tree of the C-vine, and independence copulas in all the remaining trees of the C-vine (truncated after the second tree). Figure 3 depicts the graphical representation of a 2-factor copula model with $d=5$ items as a 2-truncated C-vine. From the results in Joe et al. (2010) and Krupskii and Joe (2013), upper or lower tail-dependent copulas in trees 1 and 2 will lead to items that have more probability in joint upper or lower tail than would be expected with a discretized MVN.

### Combined Factor/Truncated Vine Copula Models

In this section, we combine the factor copula model with an 1-truncated vine copula to account for the residual dependence.

In an 1-truncated vine or Markov tree (if one is restricted to the first tree, that is truncation at level 1, then the result is a Markov tree dependence structure where two variables not connected by an edge are conditionally independent given the variables in the tree between them) with *d* variables, $d-1$ of the $d(d - 1)/2$ possible pairs are identified as the edges of a tree with *d* nodes corresponding to the items, i.e., there are a total of $d-1$ edges, where two connected pairs of items form an edge. Let *j* and *k* be indices for any pairs of items with $1 \le j < k \le d$. For a given vine tree structure, let $\mathcal {E}$ denote the set of edges. Each edge of $jk \in \mathcal {E}$ is represented with a bivariate copula $C_{jk}$ such that

$$\begin{aligned} \Pr (Y_j\le y_j,Y_k \le y_k)=C_{jk}\bigl (F_j(y_j),F_k(y_k)\bigr )=C_{jk}(a_{j,y_j+1},a_{k,y_k+1}). \end{aligned}$$

Since the densities of vine copulas can be factorized in terms of bivariate linking copulas and lower-dimensional margins, they are computationally tractable for high-dimensional continuous variables. Nevertheless, the cdf of *d*-dimensional vine copula lacks a closed form and requires $(d-1)$-dimensional integration (Joe, 1997). Hence, in order to derive the *d*-dimensional pmf using finite differences of the *d*-dimensional cdf (e.g., Braeken et al. 2007 or Nikoloulopoulos 2013) poses non-negligible numerical challenges. This problem has been solved by Panagiotelis et al. (2012) who decomposed the *d*-dimensional pmf into finite differences of bivariate copula cdfs. Hence, the pmf of an 1-truncated vine model takes the form

6 $$\begin{aligned} \pi _d(\textbf{y})=\prod _{j=1}^{d} \Pr (Y_j=y_j) \prod _{jk \in \mathcal {E}} \frac{ \Pr (Y_{j}=y_{j},Y_{k}=y_{k})}{\Pr (Y_j=y_j) \Pr (Y_k=y_k) }, \end{aligned}$$

where $$\Pr (Y_{j}=y_{j},Y_{k}=y_{k})= C_{jk}(a_{j,y_j+1},a_{k,y_k+1}) - C_{jk}(a_{j,y_j}, a_{k,y_k+1}) - C_{jk}(a_{j,y_j+1},a_{k,y_k}) + C_{jk}(a_{j,y_j},a_{k,y_k})$$ and $$\Pr (Y=y)=a_{j,y+1}-a_{j,y}.$$

The pmf of an 1-truncated vine copula in (6) can be used in the pmf of the factor copula model in (1) instead of the product to capture any residual dependencies. Hence, the pmf of the combined factor/truncated vine copula model takes the form

$$\begin{aligned} \pi _d(\textbf{y})=\int \prod _{j=1}^{d} \Pr \left( Y_j=y_j|\textbf{X}=\textbf{x}\right) \prod _{jk \in \mathcal {E}} \frac{ \Pr \left( Y_{j}=y_{j},Y_{k}=y_{k}|\textbf{X}=\textbf{x}\right) }{\Pr \left( Y_j=y_j|\textbf{X}=\textbf{x}\right) \Pr \left( Y_k=y_k|\textbf{X}=\textbf{x}\right) }\, \hbox {d}F_{\textbf{X}}(\textbf{x}). \end{aligned}$$

With one factor and an 1-truncated vine given the latent variable $X_1$ (hereafter 1-factor tree), let $C_{jk;X_1}$ be a bivariate copula such that

$$\begin{aligned} \Pr (Y_j\le y_j,Y_k\le y_k|X_1=x_1)=C_{jk;X_1}\bigl (F_{j|X_1}(y_j|x_1),F_{k|X_1}(y_k|x_1)\bigr ), \end{aligned}$$

where $F_{j|X_1}$ and $F_{k|X_1}$ are given in (2). Here, we are making the simplifying assumption that the conditional copula for the univariate distributions $F_{j|X_1}$ and $F_{k|X_1}$ does not depend on $x_1$; this is a model assumption as by Sklar’s theorem there exist such bivariate copulas that in general depend on $x_1\in [0,1]$. Then, for a given 1-truncated vine structure with a set of edges $\mathcal {E}$, the pmf of the 1-factor tree copula model is

7 $$\begin{aligned} \pi _d(\textbf{y})=\int _0^1 \prod _{j=1}^{d} f_{j|X_1}\left( y_j | x\right) \prod _{jk \in \mathcal {E}} \frac{ f_{jk|X_1} (y_j,y_k|x_1)}{ f_{j|X}\left( y_j |x \right) f_{k|X}\left( y_k |x \right) } \, \hbox {d}x, \end{aligned}$$

where

$$\begin{aligned} f_{jk|X_1}(y_j,y_k|x_1)= & {} C_{jk;X_1} \bigl ( F_{j|X_1}^+, F_{k|X_1}^+\bigr ) - C_{jk;X_1} \bigl ( F_{j|X_1}^-, F_{k|X_1}^+\bigr ) \\{} & {} - C_{jk;X_1} \bigl ( F_{j|X_1}^+, F_{k|X_1}^-\bigr ) + C_{jk;X_1} \bigl ( F_{j|X_1}^-, F_{k|X_1}^-\bigr ) \end{aligned}$$

and $f_{j|X}\left( y_j |x \right)$, $f_{k|X}\left( y_k |x \right)$ are given in (3). In the above, $F_{j|X_1}^+=F_{j|X_1}(y|x)$ and $F_{j|X_1}^-=F_{j|X_1}(y-1|x)$.

**Fig. 4:**
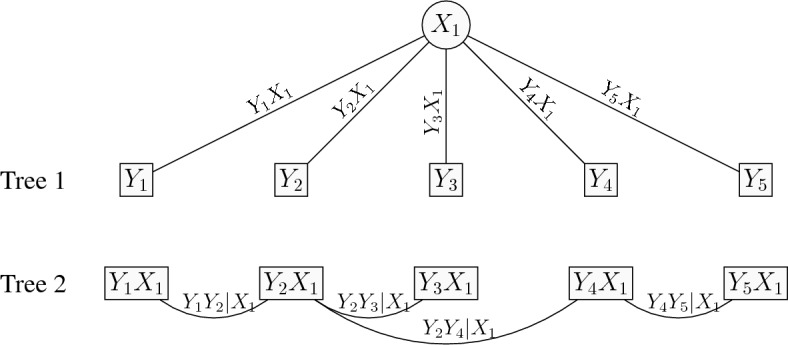
Graphical representation of a 1-factor tree copula model with $d=5$ items. The first tree is the 1-factor model. The residual dependence is captured in Tree 2 with an 1-truncated vine model.

The 1-factor tree copula model can be explained as a 2-truncated vine copula model. For the *d*-dimensional 1-factor tree copula model, the pairs at tree 1 are $Y_jX_1$ for $j=1,\ldots ,d$, the pairs at tree 2 are $Y_{j}Y_k|X_1$ for $jk\in \mathcal {E}$, and for higher trees the (conditional) copula pairs are set to independence. That is the 1-factor tree copula model has *d* bivariate copulas $C_{X_1j}$ that link $Y_j,\,j=1,\ldots ,d$ with $X_1$ in the first tree of the vine, $d-1$ bivariate copulas $C_{jk;X_1}$ that link $Y_j$ with $Y_k$ given $X_1$ in the second tree of the vine, and independence copulas in all the remaining trees of the vine (truncated after the second tree). From the results in Joe et al. (2010) and Krupskii and Joe (2013), upper or lower tail-dependent copulas in trees 1 and 2 will lead to items that have more probability in joint upper or lower tail than would be expected with a discretized MVN. Figure 4 depicts the graphical representation of a 1-factor tree copula model with $d=5$ items as a 2-truncated vine. Tree 1 shows the typical 1-factor model, while tree 2 accounts for the residual dependence by the pairwise conditional dependencies of two items conditioned on the factor $X_1$.

With two factors and an 1-truncated vine given the latent variables $X_1,X_2$ (hereafter 2-factor tree), let $C_{jk;X_1,X_2}$ be a bivariate copula cdf such that

$$\begin{aligned} \Pr (Y_j\le y_j,Y_k\le y_k|X_1,X_2)=C_{jk;X_1X_2}\bigl (F_{X_2j|X_1}(x_2,y_j|x_1),F_{X_2k|X_1}(x_2,y_k|x_1)\bigr ), \end{aligned}$$

where $F_{X_2j|X_1}$ and $F_{X_2k|X_1}$ are given in (4). Here, we are making the simplifying assumption that the conditional copula for the univariate distributions $F_{X_2j|X_1}$ and $F_{X_2k|X_1}$ does not depend on $x_1$; this is a model assumption as by Sklar’s theorem there exist such bivariate copulas that in general depend on $x_1\in [0,1]$. Then, for a given 1-truncated vine structure with a set of edges $\mathcal {E}$, the pmf of the 2-factor tree copula model is

8 $$\begin{aligned} \pi _d(\textbf{y})=\int _0^1\int _0^1 \prod _{j=1}^{d} f_{X_2j|X_1}\left( x_2,y_j | x_1\right) \prod _{jk \in \mathcal {E}} \frac{f_{jk|X_1X_2}(y_j,y_k|x_1,x_2)}{ f_{X_2j|X_1}\left( x_2,y_j |x_1 \right) f_{X_2k|X_1}\left( x_2,y_k |x_1 \right) } \, \hbox {d}{x_1} \hbox {d}{x_2}, \end{aligned}$$

where

$$\begin{aligned} f_{jk|X_1X_2}\bigl (y_j,y_k|x_1,x_2)&= C_{jk;X_1,X_2} \bigl (F_{X_2j|X_1}^+, F_{X_2k|X_1}^+\bigr ) - C_{jk;X_1,X_2} \bigl ( F_{X_2j|X_1}^-, F_{X_2k|X_1}^+\bigr ) \nonumber \\&\quad - C_{jk;X_1,X_2} \bigl (F_{X_2j|X_1}^+,F_{X_2k|X_1}^-\bigr ) + C_{jk;X_1,X_2} \bigl ( F_{X_2j|X_1}^-,F_{X_2k|X_1}^-\bigr ) \end{aligned}$$

and $f_{X_2j|X_1}(x_2,y_j|x_1)$, $f_{X_2k|X_1}(x_2,y_k|x_1)$ are as in (5). In the above $F_{X_2j|X_1}^+=F_{X_2j|X_1}(x_2,y|x_1)$ and $F_{X_2j|X_1}^-=F_{X_2j|X_1}(x_2,y-1|x_1)$.

**Fig. 5:**
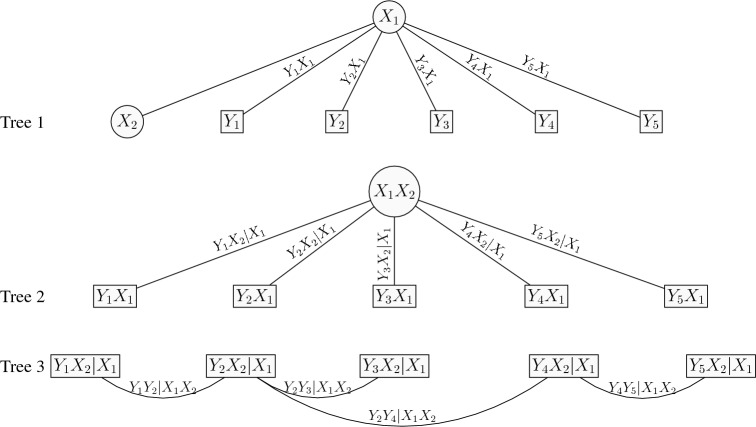
Graphical representation of a 2-factor tree copula model with $d=5$ items. The first and second trees represent the 2-factor model. The residual dependence is captured in Tree 3 with an 1-truncated vine model. Note that the factors are linked to one another with an independent copula in Tree 1.

The 2-factor tree copula model can be explained as a 3-truncated vine. For the *d*-dimensional 2-factor tree copula model, the pairs at tree 1 are $Y_jX_1$ for $j=1,\ldots ,d$, the pairs at tree 2 are $Y_jX_2|X_1$ for $j=1,\ldots ,d$, the pairs at tree 3 are $Y_{j}Y_k|X_1X_2$ for $jk\in \mathcal {E}$, and for higher trees the (conditional) copula pairs are set to independence. That is the 2-factor tree copula model has *d* bivariate copulas $C_{X_1j}$ that link $Y_j,\,j=1,\ldots ,d$ with $X_1$ in the first tree of the vine, *d* bivariate copulas $C_{X_2j}$ that link $Y_j,\,j=1,\ldots ,d$ with $X_2$ given $X_1$ in the second tree of the vine, $d-1$ bivariate copulas $C_{jk;X_1X_2}$ that link $Y_j$ with $Y_k$ given $X_1$ and $X_2$, in the third tree of the vine, and independence copulas in all the remaining trees of the vine (truncated after the third tree). From the results in Joe et al. (2010) and Krupskii and Joe (2013), upper or lower tail dependent copulas in trees 1, 2 and 3 will lead to items that have more probability in joint upper or lower tail than would be expected with a discretized MVN. Figure 5 depicts the graphical representation of a 2-factor tree copula model with $d=5$ items as a 3-truncated vine. Trees 1 and 2 show the 2-factor copula model, while tree 3 involves the pairwise conditional dependencies of two items given the factors.

For parametric 1-factor and 2-factor tree copula models, we let $C_{X_1j}$, $C_{X_2j}$ and $C_{jk;\textbf{X}}$ be parametric bivariate copulas, say with parameters $\theta _{1j}$, $\theta _{2j}$, and $\delta _{jk}$, respectively. For the set of all parameters, let $\varvec{\theta }=\{a_{jk}, \theta _{1j}, \delta _{jk}: j=1,\ldots ,d; k=1,\ldots ,K-1;jk\in \mathcal {E} \}$ for the 1-factor tree copula model and $\varvec{\theta }=\{a_{jk}, \theta _{1j}, \theta _{2j}, \delta _{jk}: j=1,\ldots ,d; k=1,\ldots ,K-1;jk\in \mathcal {E}\}$ for the 2-factor tree copula model.

### Choices of Parametric Bivariate Copulas

In line with Nikoloulopoulos and Joe (2015), we use bivariate parametric copulas that can be used when considering latent maxima, minima or mixtures of means. For different dependent items based on latent maxima or minima, multivariate extreme value and copula theory (e.g., Joe 1997) can be used to select suitable copulas that link observed to latent variables. Copulas that arise from extreme value theory have more probability in one joint tail (upper or lower) than expected with a discretized MVN distribution or a MVN copula with discrete margins. If item responses are based on discretizations of latent variables that are means, then it is possible that there can be more probability in both the joint upper and joint lower tail, compared with discretized MVN models. This happens if the respondents consist of a ‘mixture’ population (e.g., different locations or genders). From the theory of elliptical distributions and copulas (e.g., McNeil et al. 2005), it is known that the multivariate Student-*t* distribution as a scale mixture of MVN has more dependence in the tails. Extreme value and elliptical copulas can model item response data that have reflection asymmetric and symmetric dependence, respectively.

A bivariate copula *C* is reflection symmetric if its density satisfies $c(u_1,u_2)=c(1-u_1,1-u_2)$ for all $0\le u_1,u_2\le 1$. Otherwise, it is reflection asymmetric often with more probability in the joint upper tail or joint lower tail. Upper tail dependence means that $c(1-u,1-u)=O(u^{-1})$ as $u\rightarrow 0$ and lower tail dependence means that $c(u,u)=O(u^{-1})$ as $u\rightarrow 0$. If $(U_1,U_2)\sim C$ for a bivariate copula *C*, then $(1-U_1,1-U_2)\sim \widehat{C}$, where $\widehat{C}(u_1,u_2)=u_1+u_2-1+C(1-u_1,1-u_2)$ is the survival or reflected copula of *C*; this “reflection" of each uniform *U*(0, 1) random variable about 1/2 changes the direction of tail asymmetry. Choices of copulas with upper or lower tail dependence are better if the items have more probability in joint lower or upper tail than would be expected with the BVN copula. This can be shown with summaries of polychoric correlations in the upper and lower joint tail (Kadhem and Nikoloulopoulos, 2021).

After briefly providing definitions of tail dependence and reflection symmetry/asymmetry, we provide below the bivariate copula choices we consider:

- The elliptical bivariate normal (BVN) copula with cdf
  $$\begin{aligned} C(u_1,u_2;\theta )=\Phi _2\Bigl (\Phi ^{-1}(u_1;\nu ),\Phi ^{-1}(u_2;\nu );\theta \Bigr ),-1\le \theta \le 1, \end{aligned}$$
  where $\Phi$ is the univariate standard normal cdf and $\Phi _2$ is the cdf of a BVN distribution with correlation parameter $\theta$. A model with BVN copulas has latent (ordinal) variables that can be considered as (discretized) means, and there is less probability in both the joint upper and joint lower tail as the BVN copula has reflection symmetry and tail independence.
- The extreme value Gumbel copula with cdf
  $$\begin{aligned} C(u_1,u_2;\theta )=\exp \Bigl [-\Bigl \{(-\log u_1)^{\theta } +(-\log u_2)^{\theta }\Bigr \}^{1/\theta }\Bigr ], \theta \ge 1. \end{aligned}$$
  A model with bivariate Gumbel copulas has latent (ordinal) variables that can be considered as (discretized) maxima, and there is more probability in the joint upper tail as the Gumbel copula has reflection asymmetry and upper tail dependence.
- The survival Gumbel (s.Gumbel) copula with cdf
  $$\begin{aligned} C(u_1,u_2;\theta )= & {} u_1+u_2-1 + \exp \Bigl [-\Bigl \{\bigl (-\log (1-u_1)\bigr )^{\theta } +\bigl (-\log (1-u_2)\bigr )^{\theta }\Bigr \}^{1/\theta }\Bigr ], \\{} & {} \theta \ge 1. \end{aligned}$$
  A model with bivariate s.Gumbel copulas has latent (ordinal) variables that can be considered as (discretized) minima, and there is more probability in the joint lower tail as the s.Gumbel copula has reflection asymmetry and lower tail dependence.
- The elliptical bivariate $t_\nu$ copula with cdf
  $$\begin{aligned} C(u_1,u_2;\theta )=\mathcal {T}_2\Bigl (\mathcal {T}^{-1}(u_1;\nu ),\mathcal {T}^{-1}(u_2;\nu );\theta ,\nu \Bigr ),\quad -1\le \theta \le 1, \end{aligned}$$
  where $\mathcal {T}(;\nu )$ is the univariate Student-*t* cdf with (non-integer) $\nu$ degrees of freedom, and $\mathcal {T}_2$ is the cdf of a bivariate Student-*t* distribution with $\nu$ degrees of freedom and correlation parameter $\theta$. A model with bivariate $t_\nu$ copulas has latent (ordinal) variables that can be considered as mixtures of (discretized) means, since the bivariate Student-*t* distribution arises as a scale mixture of bivariate normals. A small value of $\nu$, such as $1 \le \nu \le 5$, leads to a model with more probabilities in the joint upper and joint lower tails compared with the BVN copula as the $t_\nu$ copula has reflection symmetric upper and lower tail dependence.

For the residual dependence part of the model in addition to the aforementioned bivariate parametric copulas for computational improvements, we can use the Archimedean Frank copula with cdf

$$\begin{aligned} C(u_1,u_2;\theta )=-\theta ^{-1}\log \left\{ 1+\frac{(e^{-\theta u_1}-1)(e^{-\theta u_2}-1)}{e^{-\theta }-1} \right\} , \theta \in (-\infty ,\infty )\setminus \{0\}, \end{aligned}$$

reflection symmetry and tail independence. Its tail independence is not a distributional concern about the tail dependence/asymmetry between the items due to the main result in Joe et al. (2010): for all the bivariate margins to have more probability in the joint lower or upper tail, it only suffices that the bivariate copulas in the first trees (factor part) to have upper/lower tail dependence and is not necessary for the bivariate copulas in the higher trees (residual dependence part) to have tail dependence. For discrete data, such as item response, the Frank copula has the same tail behavior with the BVN copula but provides simplified computations as it has a closed from cdf and thus, it can be preferred over the BVN copula for the residual dependence part of the model that involves finite differences of bivariate copula cdfs.

In our candidate set, families that have different strengths of tail behavior are included. These families are sufficient to account for tail asymmetry in item response data. Nikoloulopoulos and Karlis (2008) have shown that it is hard to choose a copula with similar properties from real data, since copulas with similar (tail) dependence properties provide similar fit.

## Estimation

With sample size *n* and data $\textbf{y}_1,\ldots ,\textbf{y}_n$, the joint log-likelihood of the factor tree copula models is

9 $$\begin{aligned} \ell (\varvec{\theta };\textbf{y}_1,\ldots ,\textbf{y}_n)=\sum _{i=1}^n\log \pi _d (\textbf{y}_i;\varvec{\theta }), \end{aligned}$$

with $\pi _d(\textbf{y})$ as defined in (7) and (8) for the 1-factor and 2-factor tree copula model, respectively. Maximization of (9) is numerically possible but time-consuming for large *d* because of many univariate cutpoints and dependence parameters. Hence, we approach estimation using the two-step IFM method proposed by Joe (2005) that can efficiently, in the sense of computing time and asymptotic variance, estimate the model parameters.

In the first step, the cutpoints are estimated using the univariate sample proportions. The univariate cutpoints for the *j*th item are estimated as $\hat{a}_{j,k} = \sum _{y=0}^{k} p_{j,y}$, where $p_{j,y}\,,y=0,\ldots ,K-1$ for $j=1,\ldots ,d$ are the univariate sample proportions. In the second step of the IFM method, the joint log-likelihood in (9) is maximized over the copula parameters with the cutpoints fixed as estimated at the first step. The estimated copula parameters can be obtained by using a quasi-Newton (Nash, 1990) method applied to the logarithm of the joint likelihood.

For the 1-factor tree copula model, numerical evaluation of the joint pmf can be achieved with the following steps:

1. Calculate Gauss–Legendre quadrature (Stroud and Secrest, 1966) points $\{x_q: q=1,\ldots ,n_q\}$ and weights $\{w_q: q=1,\ldots ,n_q\}$ in terms of standard uniform.
2. Numerically evaluate the joint pmf in (7) via the following approximation:
  $$\begin{aligned} \sum _{q=1}^{n_q} w_{q} \prod _{j=1}^{d} f_{j}(y_j | x_q) \prod _{jk \in \mathcal {E}} \frac{f_{jk|X_1} (y_j,y_k|x_q)}{ f_{j|X}(y_j |x_q ) f_{k|X}(y_k |x_q)}. \end{aligned}$$

For the 2-factor tree copula model, numerical evaluation of the joint pmf can be achieved with the following steps:

1. Calculate Gauss–Legendre quadrature (Stroud and Secrest, 1966) points $\{x_{q_1}: q_1 = 1,\ldots ,n_q\}$ and $\{x_{q_2}: q_2 = 1,\ldots ,n_q\}$ and weights $\{w_{q_1}: q_1 = 1,\ldots ,n_q\}$ and $\{w_{q_2}: q_2 = 1,\ldots ,n_q\}$ in terms of standard uniform.
2. Numerically evaluate the joint pmf in (8) via the following approximation in a double sum:
  $$\begin{aligned} \sum _{q_1=1}^{n_q} \sum _{q_2=1}^{n_q} w_{q_1} w_{q_2} \prod _{j=1}^{d} f_{X_2j|X_1}(x_{q_2},y_j | x_{q_1}) \prod _{ jk \in \mathcal {E}} \frac{f_{jk|X_1X_2}(y_j,y_k|x_{q_1},x_{q_2})}{f_{X_2j|X_1}(x_{q_2},y_j|x_{q_1}) f_{X_2k|X_1}(x_{q_2},y_k |x_{q_1} )}. \end{aligned}$$

With Gauss–Legendre quadrature, the same nodes and weights are used for different functions; this helps in yielding smooth numerical derivatives for numerical optimization via quasi-Newton. Our comparisons show that $n_q=25$ quadrature points are adequate with good precision.

## Model Selection

In this section, we will discuss model selection strategies for the factor tree copula models. Section 3.1 proposes tree structure selection methods for the residual dependence part of the model that assume the factor tree copula models are constructed with BVN copulas. Section 3.2 proposes a heuristic algorithm that sequentially selects suitable bivariate copulas to account for any tail dependence/asymmetry. Similar heuristics have been successfully used for selecting suitable bivariate copulas to account for any tail dependence/asymmetry in factor (Kadhem and Nikoloulopoulos, 2021) and structured factor (Kadhem and Nikoloulopoulos, 2023a) copula models.

### 1-Truncated Vine Tree Structure Selection

The 1-truncated vine tree structure is unknown; hence, it has to be determined. The number of different possible 1-truncated vines in *d* dimensions is large. Hence, we need a way of selecting a reasonable tree. Following earlier contributions on the model selection of truncated vine copula models (e.g., Brechmann et al. 2012), we will heuristically proceed by modeling the most strong dependencies in the tree and construct a tree on *d* nodes corresponding to the *d* variables, where all nodes are connected by a common edge, that is, have $d-1$ neighbors. These edges have a weight according to a measure of pairwise dependence, say $r_{jk}$, between the respective two variables. We find the maximum spanning tree, which is a tree on all nodes that maximizes the pairwise dependencies, using the well-known algorithm of Prim (1957). That is we find the tree with $d-1$ edges $\mathcal {E}$ that minimizes $\sum _{\mathcal {E}}\log (1-r_{jk}^2).$ The minimum spanning tree algorithm of Prim (1957) guarantees to find the optimal solution when edge weights between nodes $1\le k<j\le d$ are given by $\log (1-r_{jk}^2)$.

We use two different measures of pairwise dependence. The first measure is the estimated polychoric correlation (Olsson, 1979). The sample polychoric correlation for all possible pairs of items can be estimated as

$$\begin{aligned}&\hat{\rho }_{jk}=\text{ argmax}_\rho \sum _{i=1}^n\log \Bigl (\Phi _2(\alpha _{j,y_{ij}+1},\alpha _{k,y_{ik}+1}; \rho )-\Phi _2(\alpha _{j,y_{ij}+1},\alpha _{k,y_{ik}}; \rho )\nonumber \\ {}&\quad - \Phi _2(\alpha _{j,y_{ij}},\alpha _{k,y_{ik}+1}; \rho )+\Phi _2(\alpha _{j,y_{ij}},\alpha _{k,y_{ik}}; \rho )\Bigr ),\quad 1\le j<k\le d, \end{aligned}$$

where $\Phi _2(\cdot ,\cdot ;\rho )$ is the BVN cdf with correlation parameter $\rho$.

The second measure of pairwise dependence that we exploit is based on the *p*-factor copula models with BVN copulas. When all the bivariate copulas are BVN, the *p*-factor copula model is the same as the discretized MVN model with a *p*-factor correlation matrix, also known as the *p*-dimensional normal ogive model (Nikoloulopoulos and Joe, 2015). The 1-factor copula model with BVN copulas is the same as the variant of Samejima’s (1969) graded response IRT model, known as normal ogive model (McDonald, 1997) with a 1-factor correlation matrix $R=(r_{jk})$ with $r_{jk}=\theta _{1j}\theta _{1k}$ for $j\ne k$. The 2-factor copula model with BVN copulas is the same as the bidimensional (2-factor) normal ogive model with a 2-factor correlation matrix $R=(r_{jk})$ with $r_{jk}=\theta _{1j}\theta _{1k}+ \theta _{2j}\theta _{2k}[(1-\theta _{1j}^2)(1-\theta _{1k}^2)]^{1/2}$ for $j\ne k$. The parameter $\theta _{1j}$ of $C_{X_1j}$ is the correlation of the underlying normal variable $Z_j$ of $Y_j$ with $Z_{01}=\Phi ^{-1}(X_1)$, and the parameter $\theta _{2j}$ of $C_{X_2j}$ is the partial correlation between $Z_j$ and $Z_{02}=\Phi ^{-1}(X_1)$ given $Z_{01}$. Subsequently, for all possible pair of items we can estimate the partial correlations between $Z_j$ and $Z_k$ given $Z_{01}$ and between $Z_j$ and $Z_k$ given $Z_{01},Z_{02}$ via the relations

$$\begin{aligned} \hat{\rho }_{jk;Z_{01}} = \frac{\hat{\rho }_{jk} -\hat{\theta }_{1j} \hat{\theta }_{1k} }{ \sqrt{ (1-\hat{\theta }_{1j}^2) (1-\hat{\theta }_{1k}^2) } } \quad \text{ and }\quad \hat{\rho }_{jk;Z_{01},Z_{02}} = \frac{ \hat{\rho }_{jk;Z_{01}} - \hat{\theta }_{2j} \hat{\theta }_{2k} }{ \sqrt{ (1-\hat{\theta }_{2j}^2) (1-\hat{\theta }_{2k}^2) } }, \end{aligned}$$

respectively, where $\hat{\theta }_{1j},\hat{\theta }_{1k}$ are the estimated unidimensional normal ogive model’s parameters and $\hat{\theta }_{1j},\hat{\theta }_{1k},\hat{\theta }_{2j},\hat{\theta }_{2k}$ are the estimated bidimensional normal ogive model’s parameters. We refer to Nikoloulopoulos and Joe (2015, Sect. 2.3) for further details and explanations on the normal ogive models as special cases of factor copula models.

We call polychoric and partial correlation selection algorithm when the pairwise dependencies are the polychoric and partial correlations, respectively. The polychoric correlation selection algorithm selects the edges $\mathcal {E}$ of the tree that minimize the sum of the weights $\log (1-\hat{\rho }_{jk}^2)$, while the partial correlation selection algorithm the sum of the weights $\log (1-\hat{\rho }_{jk;Z_{01}}^2)$ for the 1-factor tree copula model and $\log (1-\hat{\rho }_{jk;Z_{01},Z_{02}}^2)$ for the 2-factor tree copula model.

### Bivariate Copula Selection

We propose a heuristic method that selects appropriate bivariate copulas for the proposed models. It starts with an initial assumption that all bivariate copulas are BVN and independent copulas in the factor and 1-truncated vine copula model, respectively. Then, sequentially suitable copulas with lower or upper tail dependence are assigned where necessary to account for more probability in one or both joint tails. For ease of interpretation, we do not mix Gumbel, s.Gumbel, $t_\nu$ and BVN for a single tree of the model; e.g., for the 2-factor tree copula model we allow three different copula families, one for the first factor, one for the second factor and one for the 1-truncated vine (residual dependence part of the model).

The selection algorithm involves the following steps:

1. Start with a factor tree copula model with BVN and independent copulas in the factor and 1-truncated vine copula parts of the model, respectively.
2. Factor part
  - Factor 1
    i. Fit all the possible models, iterating over all the bivariate copula candidates that link each of the items to $X_1$.
    ii. Select the bivariate copula that corresponds to the highest log-likelihood.
    iii. Replace the BVN with the selected bivariate copula that links each of the items to $X_1$.
  - Factor 2
    i. Fit all the possible models, iterating over all the copula candidates that link each of the items to $X_2$.
    ii. Select the bivariate copula that corresponds to the highest log-likelihood.
    iii. Replace BVN with the selected bivariate copula that links each of the items to $X_2$.
3. 1-truncated vine part
  - Select the best 1-truncated vine tree structure $\mathcal {E}$ using both the polychoric and partial correlation selection algorithms proposed in Sect. 3.1.
  - Fit all the possible models, iterating over all the bivariate copula candidates that link the pairs of items $\in \mathcal {E}$ given the factors.
  - Select the bivariate copula that corresponds to the highest log-likelihood.
  - Replace the independence copula with the selected bivariate copula that links each pair of items $\in \mathcal {E}$ given the factors.

## Simulations

Extensive simulation studies are conducted to assess the (a) efficiency of the proposed estimation method, (b) performance of the model selection algorithms to select the correct 1-truncated vine tree structure for the residual dependence part of the model and (c) reliability of using the heuristic algorithm to select the true (simulated) bivariate linking copulas.

We randomly generated 1, 000 datasets with sample size $n = 500$ and $d=\{8,16,24\}$ items with $K=5$ equally weighted categories from an 1-factor and 2-factor tree copula models with Gumbel copulas. The items in the last tree are either serially connected in ascending order with an 1-truncated D-vine or randomly connected with a 1-truncated regular vine.

For the Gumbel copulas, we set the copula parameters in Kendall’s $\tau$ scale via the functional relation,

10 $$\begin{aligned} \tau (\theta )=1-\theta ^{-1}. \end{aligned}$$

We use $\tau$’s in equally spaced sequences, i.e., $\tau (\theta _{1j},\,j=1,\ldots ,d)=\{0.70,\ldots ,0.40 \}$ and $\tau (\theta _{2j},\,\,j=1,\ldots ,d)=\{0.55,\ldots ,0.25 \}$ for the factor copula parts of the models and $\tau (\delta _{jk},\,jk\in \mathcal {E})=\{0.40,\ldots ,0.10 \}$ for the 1-truncated vine copula part of the model for the 1-factor and 2-factor tree copula model, respectively.

Tables 1 and 2 present the resulting biases, standard deviations (SD) and root mean square errors (RMSE), scaled by *n*, from the simulations of the 1-factor and 2-factor tree copula models with Gumbel copulas, respectively, and an 1-truncated D-vine residual dependence structure. The results indicate that the proposed approximation method is efficient for estimating the factor tree copula models and the efficiency improves as the dimension increases.

**Fig. 6:**
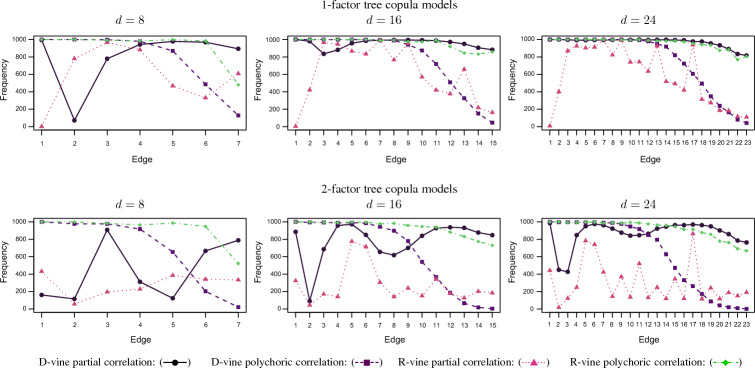
Small sample of size $n = 500$ simulations ($10^3$ replications) and $d=\{8,16,24\}$ items with $K=5$ equally weighted categories from 1-factor and 2-factor tree copula models with Gumbel copulas and an 1-truncated D- or regular (R) vine residual dependence structure and resultant number of times a pair of items is correctly selected as an edge for each of the edges of the 1-truncated D- or R-vine copula for both the partial and polychoric correlation selection algorithms.

In Fig. 6, we report the frequency of a pair of items is correctly selected as an edge for each of the edges of the 1-truncated vine from the simulations of the 1- and 2-factor tree copula models with Gumbel copulas with $d=8$, $d=16$ and $d=24$ items for both the partial and polychoric correlation selection algorithms. It has been shown that the partial correlation selection algorithm as the dimension increases performs extremely well for the 1-truncated D-vine residual dependence structure, but poorly for the 1-truncated regular vine structure. The quite contrary (or complimentary) results are seen for the polychoric correlation selection algorithm. The polychoric correlation selection algorithm rather performs extremely well in selecting the true edges in the 1-truncated regular vine residual dependence structure. It is most accurate for the initial edges, while it is less accurate for the final edges. This is because the dependence strength is represented in descending order as $\tau =\{0.40,\ldots ,0.10 \}$, so the polychoric correlation selection algorithm is highly reliable to select the edges with stronger dependence. The edges with weaker dependence are not easily quantified and can be approximated with other edges that lead to a similar correlation matrix or even accounted for by the previous trees (factor copula models).

**Table 1:** Small sample of size $n = 500$ simulations ($10^3$ replications) and $d=\{8,16,24\}$ items with $K=5$ equally weighted categories from an 1-factor tree copula model with Gumbel copulas and an 1-truncated D-vine residual dependence structure for $d=\{8, 16, 24\}$ and resultant biases, root mean square errors (RMSE), and standard deviations (SD), scaled by *n*, for the IFM estimates.

$d=8$	1st tree (1-factor copula)								2nd tree (1-truncated D-vine copula)							
$\tau$	0.70	0.66	0.61	0.57	0.53	0.49	0.44	0.40	0.40	0.35	0.30	0.25	0.20	0.15	0.10	
*n*Bias	6.19	5.83	8.34	7.30	4.13	$-$0.46	$-$2.47	$-$2.77	$-$14.23	$-$16.11	$-$15.79	$-$9.90	$-$2.86	1.19	1.42	
*n*SD	20.48	21.24	19.05	17.56	16.43	16.56	15.79	16.05	44.97	33.61	28.66	25.17	21.68	19.87	18.54	
*n*RMSE	21.40	22.03	20.80	19.01	16.94	16.57	15.98	16.29	47.17	37.27	32.72	27.05	21.87	19.91	18.60	

$d=16$	1st tree (1-factor copula)															
$\tau$	0.70	0.68	0.66	0.64	0.62	0.60	0.58	0.56	0.54	0.52	0.50	0.48	0.46	0.44	0.42	0.40
*n*Bias	2.76	3.43	5.22	6.18	6.02	4.66	2.96	2.19	0.79	0.20	0.05	$-$1.43	$-$1.74	$-$1.02	$-$1.80	$-$0.93
*n*SD	10.89	11.31	11.85	11.94	12.08	11.91	12.35	12.45	12.65	13.26	12.96	13.66	13.66	14.51	14.55	14.19
*n*RMSE	11.23	11.81	12.95	13.45	13.49	12.79	12.70	12.64	12.68	13.26	12.96	13.74	13.77	14.55	14.66	14.22

	2nd tree (1-truncated D-vine copula)															
$\tau$	0.40	0.38	0.36	0.34	0.31	0.29	0.27	0.25	0.23	0.21	0.19	0.16	0.14	0.12	0.10	
*n*Bias	$-$6.55	$-$9.58	$-$12.27	$-$11.32	$-$9.85	$-$6.42	$-$4.51	$-$2.46	$-$1.01	0.46	0.70	1.35	1.96	1.17	1.59	
*n*SD	22.62	22.71	21.92	20.66	19.36	18.59	18.95	18.22	17.92	18.02	17.21	17.20	16.79	16.91	16.62	
*n*RMSE	23.55	24.65	25.12	23.56	21.72	19.67	19.48	18.39	17.95	18.02	17.22	17.25	16.90	16.95	16.70	

$d=24$	1st tree (1-factor copula)															
$\tau$	0.70	0.69	0.67	0.66	0.65	0.63	0.62	0.61	0.60	0.58	0.57	0.56	0.54			
*n*Bias	1.61	1.89	3.41	4.20	4.35	3.84	3.13	2.52	2.29	1.68	1.03	0.44	$-$0.21			
*n*SD	9.72	10.39	10.86	11.06	11.13	10.86	11.28	11.32	11.61	11.99	11.76	11.90	12.10			
*n*RMSE	9.86	10.56	11.38	11.83	11.95	11.52	11.70	11.59	11.83	12.11	11.80	11.91	12.11

**Table 2:** Small sample of size $n = 500$ simulations ($10^3$ replications) and $d=24$ items with $K=5$ equally weighted categories from a 2-factor tree copula model with Gumbel copulas and an 1-truncated D-vine residual dependence structure and resultant biases, root mean square errors (RMSE), and standard deviations (SD), scaled by *n*, for the IFM estimates.

	1st tree (1st factor of 2-factor copula)											
$\tau$	0.70	0.69	0.67	0.66	0.65	0.63	0.62	0.61	0.60	0.58	0.57	0.56
*n*Bias	$-$5.74	$-$3.26	$-$0.07	2.35	3.96	4.12	3.60	3.94	4.05	3.73	4.58	4.27
*n*SD	26.55	26.96	27.90	27.43	25.80	24.89	25.15	24.57	23.62	23.93	23.89	23.53
*n*RMSE	27.16	27.15	27.90	27.53	26.11	25.23	25.41	24.89	23.97	24.22	24.33	23.91

	1st tree (1st factor of 2-factor copula, continued)											
$\tau$	0.54	0.53	0.52	0.50	0.49	0.48	0.47	0.45	0.44	0.43	0.41	0.40
*n*Bias	3.74	4.83	4.17	5.08	4.28	4.56	5.15	4.80	4.82	4.05	4.42	2.96
*n*SD	23.21	23.04	22.38	23.15	22.39	23.75	22.93	22.04	22.38	21.99	22.71	21.74
*n*RMSE	23.51	23.54	22.77	23.70	22.80	24.18	23.50	22.56	22.89	22.36	23.14	21.94

	2nd tree (2nd factor of 2-factor copula)											
$\tau$	0.55	0.54	0.52	0.51	0.50	0.48	0.47	0.46	0.45	0.43	0.42	0.41
*n*Bias	4.31	1.24	2.81	0.39	$-$0.58	$-$1.81	$-$2.58	$-$3.06	$-$6.03	$-$6.58	$-$8.23	$-$9.13
*n*SD	40.65	41.80	42.93	45.05	43.16	42.69	41.67	40.68	40.38	41.00	41.35	39.73
*n*RMSE	40.88	41.82	43.02	45.05	43.17	42.73	41.75	40.79	40.83	41.52	42.16	40.76

	2nd tree (2nd factor of 2-factor copula, continued)											
$\tau$	0.39	0.38	0.37	0.35	0.34	0.33	0.32	0.30	0.29	0.28	0.26	0.25
*n*Bias	$-$9.58	$-$12.73	$-$13.14	$-$11.90	$-$9.67	$-$10.48	$-$12.89	$-$11.57	$-$11.57	$-$12.77	$-$11.14	$-$8.04
*n*SD	41.24	41.35	40.48	40.60	41.84	42.41	40.90	38.62	40.15	37.78	39.96	38.41
*n*RMSE	42.34	43.27	42.56	42.31	42.94	43.68	42.88	40.31	41.78	39.88	41.49	39.25

	3rd tree (1-truncated D-vine copula)											
$\tau$	0.40	0.39	0.37	0.36	0.35	0.33	0.32	0.30	0.29	0.28	0.26	0.25
*n*Bias	0.10	$-$4.49	$-$9.56	$-$10.74	$-$9.52	$-$9.21	$-$6.47	$-$4.90	$-$2.94	$-$3.25	$-$0.50	$-$0.21
*n*SD	32.64	35.17	31.46	28.61	27.74	24.35	24.49	22.53	25.08	23.54	22.79	20.38
*n*RMSE	32.64	35.46	32.88	30.56	29.33	26.03	25.33	23.06	25.25	23.76	22.80	20.38

	3rd tree (1-truncated D-vine copula, continued)											
$\tau$	0.24	0.22	0.21	0.20	0.18	0.17	0.15	0.14	0.13	0.11	0.10	
*n*Bias	0.85	1.52	2.04	0.34	1.66	1.66	1.76	2.45	2.02	2.29	2.25	
*n*SD	21.06	20.56	20.37	22.01	20.16	20.08	19.14	19.56	18.21	18.11	18.33	
*n*RMSE	21.07	20.61	20.48	22.01	20.23	20.15	19.22	19.71	18.33	18.25	18.47	

**Table 3:** Number of times each of the bivariate linking copulas was chosen over the 100 simulated datasets from the 1-factor tree copula model with Gumbel copulas at the first tree and $t_3$ copulas at the second tree with sample sizes $n = \{100,300,500\}$ and $d=\{8,16,24\}$ items with $K=\{3,5\}$ equally weighted categories.

		*n*	*d*	BVN		$t_2$		$t_3$		$t_5$		$t_7$		Gumbel		s.Gumbel	
				$K=3$	$K=5$	$K=3$	$K=5$	$K=3$	$K=5$	$K=3$	$K=5$	$K=3$	$K=5$	$K=3$	$K=5$	$K=3$	$K=5$
Tree 1	Gumbel	100	8	28	12	0	0	4	5	3	5	2	5	**62**	**73**	1	0
Tree 2	$t_3$			5	0	26	18	**15**	**43**	16	20	13	9	11	8	14	2
Tree 1	Gumbel	300		12	1	0	0	0	0	3	0	3	6	**82**	**93**	0	0
Tree 2	$t_3$			0	0	20	6	**40**	**69**	23	22	7	2	5	1	5	0
Tree 1	Gumbel	500		2	0	0	0	0	0	1	1	2	2	**95**	**97**	0	0
Tree 2	$t_3$			0	0	15	6	**64**	**83**	18	10	1	1	0	0	2	0
Tree 1	Gumbel	100	16	26	9	0	0	0	0	0	1	3	7	**71**	**83**	0	0
Tree 2	$t_3$			2	0	27	9	**36**	**62**	14	23	11	5	4	0	6	1
Tree 1	Gumbel	300		15	0	0	0	0	0	0	0	2	1	**83**	**99**	0	0
Tree 2	$t_3$			0	0	9	0	**70**	**92**	18	8	2	0	0	0	1	0
Tree 1	Gumbel	500		4	0	0	0	0	0	0	0	1	0	**95**	**100**	0	0
Tree 2	$t_3$			0	0	16	0	**76**	**97**	8	3	0	0	0	0	0	0
Tree 1	Gumbel	100	24	13	6	0	0	0	0	2	0	3	7	**82**	**87**	0	0
Tree 2	$t_3$			0	0	13	4	**55**	**72**	21	24	5	0	3	0	3	0
Tree 1	Gumbel	300		4	0	0	0	0	0	0	0	0	0	**96**	**100**	0	0
Tree 2	$t_3$			0	0	2	0	**85**	**97**	12	3	0	0	1	0	0	0
Tree 1	Gumbel	500		1	0	0	0	0	0	0	0	0	0	**99**	**100**	0	0
Tree 2	$t_3$			0	0	1	0	**90**	**98**	9	2	0	0	0	0	0	0

The items in the second tree are serially connected in ascending order with an 1-truncated D-vine. The numbers of correct choices are boldfaced.

To examine the reliability of using the heuristic algorithm to select the true (simulated) bivariate linking copulas, 100 datasets with sample sizes $n = \{100,300,500\}$ and $d=\{8,16,24\}$ items with $K=\{3,5\}$ equally weighted categories were generated from the 1-factor tree copula model with Gumbel copulas at the first tree and $t_3$ copulas at the second tree. The items in the second tree are serially connected in ascending order with an 1-truncated D-vine. We use the same true $\tau$’s as in our preceding simulation study; for the $t_\nu$ copulas, we set the copula parameters in Kendall’s $\tau$ scale via the functional relation,

11 $$\begin{aligned} \tau (\theta )=\frac{2}{\pi }\arcsin (\theta ). \end{aligned}$$

Table 3 presents the number of times each of the bivariate linking copulas was chosen over the 100 simulation runs. It is revealed that the model selection algorithm performs extremely well with different choices of linking copulas as the sample size *n*, the number of items *d* or categories *K* increases. As the number of categories decreases, the tail asymmetries of the items cannot be easily quantified; hence, bivariate parametric copulas are less distinguishable. If the true bivariate copula has distinct dependence properties (e.g., the Gumbel copula), then the algorithm selects the true copula with a high probability. Low selection rates occur for small samples/dimensions if the true copulas have similar tail dependence properties, since it is then difficult to distinguish among parametric families of copulas (Nikoloulopoulos and Karlis, 2008). For example, when the true bivariate copula is the $t_3$ the algorithm selected either $t_2$, $t_3$ or $t_5$, because $t_\nu$ copulas with a small degree of freedom $\nu$ provide similar reflection symmetric tail dependence.

Other simulations we have done with unequally weighted categories show that the algorithms are not sensitive to the threshold placement as for the ordinal marginal distributions we use the step or empirical distribution function $F_j(y)=a_{j,y+1}$ with jumps at $0,\ldots ,K-1$.

## Application

In this section, we illustrate the proposed methodology by analyzing $d=20$ items from a subsample of $n=221$ veterans who reported clinically significant Post-Traumatic Stress Disorder (PTSD) symptoms (Armour et al., 2017). The items are divided into four domains: (1) intrusions (e.g., repeated, disturbing and unwanted memories), (2) avoidance (e.g., avoiding external reminder of the stressful experience), (3) cognition and mood alterations (e.g., trouble remembering important parts of the stressful experience) and (4) reactivity alterations (e.g., taking too many risks or doing things that could cause you harm). Each item is answered in a five-point ordinal scale: “0 = Not at all," “1 = A little bit," “2 = Moderately," “3 = Quite a bit" and “4 = Extremely". The dataset and its complete description can be found in Armour et al. (2017) or in the R package **BGGM** (Williams and Mulder, 2020). With four domains, one might anticipate four factors or one factor and four residual dependence clusters. The proposed factor-tree copula models do not require a priori knowledge of obvious subgroups and form a dependence structure with conditional dependence given one or two latent variables rather than mitigating the conditional independence using four factors. The evaluation of the joint likelihood requires only low-dimensional integration, as in the 1- and 2-factor copula models while a 4-factor copula model would require 4-dimensional integration.

**Table 4:** Average observed polychoric correlations $\rho _N$ and lower/upper semi-correlations $\rho _N^-$/$\rho _N^+$ for all pairs of items for the Post-Traumatic Stress Disorder dataset, along with the corresponding theoretical correlation and semi-correlations for BVN, $t_2$, $t_5$, Frank, Gumbel, and survival Gumbel (s.Gumbel) copulas.

	$\rho _N$	$\rho _N^-$	$\rho _N^+$
Observed	0.35	0.26	0.47
BVN	0.35	0.16	0.16
$t_2$	0.35	0.49	0.49
$t_5$	0.35	0.35	0.35
Frank	0.35	0.10	0.10
Gumbel	0.35	0.11	0.37
s.Gumbel	0.35	0.37	0.11

For some items, it is plausible that a veteran might be thinking about the maximum trauma (or a high quantile) of many past events. For example, for the items in the first domain, a participant might reflect on past relevant events where an intrusion affected their life; then by considering the worst case, i.e., the event where the negative effect of an intrusion in their life was substantial, they choose an appropriate ordinal response. For some of the other items, one might consider a median or less extreme harm of past relevant events. To sum up, the items appear to be a mixed selection between discretized averages and maxima so that a factor model with more probability in the joint upper tail might be an improvement over a factor model based on a discretized MVN.

The interpretations as above suggest that a factor tree with a combination of Gumbel and BVN or $t_\nu$ copulas might provide a better fit. To further explore the above interpretations, we calculate the average of lower and upper polychoric semi-correlations (Kadhem and Nikoloulopoulos, 2023a, 2021) for all variables to check if there is any overall tail asymmetry. For comparison, we also report the theoretical semi-correlations under different choices of copulas. Choices of copulas with upper or lower tail dependence are better if the items have more probability in joint lower or upper tail than would be expected with the BVN copula. For the BVN and $t_\nu$ copulas $\rho _N^{-}=\rho _N^{+}$, while for the Gumbel and s.Gumbel copulas $\rho _N^{-}<\rho _N^{+}$ and $\rho _N^{-}>\rho _N^{+}$, respectively. The sample versions of $\rho _N^{+},\rho _N^{-}$ for item response data are the polychoric correlations in the joint lower and upper quadrants of $Y_{j}$ and $Y_{k}$ (Kadhem and Nikoloulopoulos, 2021). Table 4 shows averages of the polychoric semi-correlations $\rho _N$ for all pairs along with the theoretical upper/lower semi-correlations $\rho _N^-$/$\rho _N^+$ under different choices of copulas. Overall, we see that there is more observed polychoric correlation in the joint upper tail than the joint lower tail, i.e., $\rho _N^+=0.47>\rho _N^-=0.26$, suggesting that factor tree copula models with a combination of Gumbel and $t_5$ bivariate copulas might be plausible given that the $t_5$ copulas provide the same lower and upper tail dependence or semicorrelation, while the Gumbel copulas provide only upper tail dependence or semicorrelation. Their combination is required to model the reflection asymmetric tail dependence shown by the observed polychoric semi-correlations.

We then select a suitable 1-truncated vine tree structure using the polychoric and partial correlation selection algorithms proposed in Sect. 3.1 and compute various discrepancy measures between the observed polychoric correlation matrix $\textbf{R}_{\textrm{observed}}$ and the correlation matrix $\textbf{R}_{\textrm{model}}$ based on factor tree copula models with BVN copulas. We report the maximum absolute correlation difference $D_1=\max |\textbf{R}_{\textrm{model}} - \textbf{R}_{\textrm{observed}}|$, the average absolute correlation difference $D_2=\textrm{avg}| \textbf{R}_{\textrm{model}} - \textbf{R}_{\textrm{observed}}|$ and the correlation matrix discrepancy measure $D_3=\log \bigl ( \det (\textbf{R}_{\textrm{model}}) \bigr ) - \log \bigl ( \det (\textbf{R}_{\textrm{observed}})\bigr ) + \textrm{tr}( \textbf{R}^{-1}_{\textrm{model}} \textbf{R}_{\textrm{observed}} ) - d$. We aim to obtain a dependence structure that results in the lowest discrepancy measure; this will indicate a suitable vine structure for the item response data on hand. For a baseline comparison, we also compute the discrepancy measures for the 1- and 2-factor (tree) copula models with BVN copulas. The factor copula models with BVN copulas are equivalent to the uni- and bidimensional normal ogive models, and the factor tree copula models use the uni- and bidimensional normal ogive models as the factor parts of the models, while the residual dependence parts are discretized MVN distributions.

**Table 5:** Measures of discrepancy between the observed polychoric correlation matrix and the correlation matrix based on the 1-factor, 2-factor, 1-factor tree, and 2-factor tree copula models with BVN copulas, along with the AICs, Vuong’s 95% CIs, for the 1-factor, 2-factor, 1-factor tree, and 2-factor tree copula models with BVN and selected copulas for the Post-Traumatic Stress Disorder dataset. Alg.1: partial correlation selection algorithm; Alg. 2: polychoric correlation selection algorithm.

	Factor copula		1-factor tree copula		2-factor tree copula	
	1-factor	2-factor	Alg.1	Alg.2	Alg.1	Alg.2
*BVN copulas*						
$D_1$	0.40	0.30	0.23	0.20	0.15	0.20
$D_2$	0.08	0.05	0.05	0.05	0.03	0.05
$D_3$	4.53	2.80	1.75	1.83	1.17	1.75
#parameters	20	39	39	39	58	58
AIC	12,031.1	11,764.0	11,632.4	11,642.1	11,549.1	11,611.8
*Selected copulas*						
#parameters	20	40	39	39	59	59
AIC	11,800.4	11,413.5	11,355.3	11,344.89	11,189.1	11,240.3
Vuong’s 95% CI$^{1}$	( 0.21, 0.63)	(0.25, 0.79)	(0.37, 0.89)	(0.43, 0.91)	( 0.54, 1.09)	(0.58, 1.11)
Vuong’s 95% CI$^{2}$	(1.50, 2.31)	(0.99, 1.67)	(0.79, 1.40)	(0.83, 1.40)	–	(0.69, 1.24)
Vuong’s 95% CI$^{3}$	(1.17, 1.80)	(0.60, 1.02)	(0.30, 0.63)	(0.27, 0.61)	–	($-$0.002, 0.23)

$^{1}$Selected factor (tree) copula models versus their Gaussian analogues.

$^{2}$Selected 2-factor tree copula model with Alg.1 versus other fitted models with BVN copulas.

$^{3}$Selected 2-factor tree copula model with Alg.1 versus other fitted models with selected copulas.

After finding a suitable vine structure, we construct a plausible factor tree copula model, to analyze any type of items, by using the proposed heuristic algorithm in Sect. 3.2. We use the AIC at the IFM estimates as a rough diagnostic measure for model selection between the models. In addition, we use the Vuong (1989) procedure that is based on the sample version of the difference in Kullback–Leibler divergence. Let Model 1 and Model 2 have parametric pmfs $\pi ^{(1)}_d(\textbf{y};\widehat{\varvec{\theta }}_1)$ and $\pi ^{(2)}_d(\textbf{y};\widehat{\varvec{\theta }}_1)$, respectively; $\widehat{\varvec{\theta }}_1,\widehat{\varvec{\theta }}_2$ are the IFM estimates. The procedure computes the average $\bar{D}$ of the log differences $D_i=\log \left[ \frac{\pi ^{(2)}_d(\textbf{y}_i;\widehat{\varvec{\theta }}_2)}{\pi ^{(1)}_d(\textbf{y}_i;\widehat{\varvec{\theta }}_1)}\right]$ between the two parametric models. Vuong (1989) has shown that asymptotically $\sqrt{n}\bar{D}/s\sim N(0,1)$; $s^2=\frac{1}{n-1}\sum _{i=1}^n(D_i-\bar{D})^2$. Hence, the AIC adjusted Vuong’s 95% CI is $\bar{D} - n^{-1} [\dim (\widehat{\varvec{\theta }}_2) - \dim (\widehat{\varvec{\theta }}_1)] \pm 1.96 \times \frac{1}{\sqrt{n}} \sigma$. If it includes 0, then Model 1 and Model 2 are considered to be non-significantly different, while if it is above 0, then Model 2 is favorable and considered to fit better than Model 1. We will compare the (1) selected factor (tree) copula models (Model 2) versus their Gaussian analogues (Model 1), (2) selected factor tree copula model according to AIC (Model 2) versus all the other factor (tree) copula models with BVN copulas (Model 1), and (3) selected factor tree copula model according to AIC (Model 2) versus all the other factor (tree) copulas models with selected copulas (Model 1).

Table 5 shows that the observed polychoric correlation matrix of the data has a 2-factor tree structure according to the discrepancy measures. The table also gives the AICs and the 95% CIs of Vuong’s tests for all the fitted models. The best fitted model, based on AIC values, is the 2-factor tree copula model obtained from the partial correlation selection algorithm. From the Vuong’s 95% Cls, it is shown that 2-factor tree copula model provides a big improvement over its Gaussian analogue and outperforms all the other fitted models except the 2-factor tree obtained from the polychoric correlation selection algorithm. The tree selection algorithms might not yield into the same ‘true’ vine tree; however, closely approximated factor tree copula models are achieved.

Table 6 includes the copula parameter estimates in Kendall’s $\tau$ scale and their standard errors (SE) for the selected 2-factor and 2-factor tree copula models. The latter is obtained from the partial selection algorithm. It has the $t_2$ for the first tree, Gumbel for the second tree, and $t_5$ for the third tree. The 2-factor tree copula model is mostly constructed with $t_\nu$ bivariate copulas with a small $\nu$ which are suitable for both positive and negative dependence; however, the highest dependence is found in the second factor which is constructed with Gumbel copulas. This is in line with both the initial interpretations and preliminary analysis which suggest that some items can be considered as discretized maxima. To show the improvement of the copula models over their Gaussian analogues, we also report the 2-factor and 2-factor tree copula models with BVN copulas. The former is equivalent to the bidimensional normal ogive model and the latter uses the bidimensional normal ogive model as the factor part of the model, while the residual dependence part is a discretized MVN distribution. For the two-factor copula model with BVN copulas or bidimensional normal ogive model, one parameter for the second factor is set to zero and the likelihood is maximized with respect to other $2d-1$ parameters. We report the varimax transform of the loadings (a reparametrization of 2*d* parameters), converted to factor copula parameters via the relations in Sect. 3.1. However, using other than BVN copulas, the two-factor copula model is near-identifiable with 2*d* bivariate linking copulas, as it as been demonstrated by Krupskii and Joe (2013) and Nikoloulopoulos and Joe (2015) and no rotation is required. In terms of identifiability of signs of parameters, the factor copula model based on t$_\nu$ is like that based on BVN. If $\theta _{1j}\rightarrow -\theta _{1j}$, $j=1,\ldots ,d$ or if $\theta _{2j}\rightarrow -\theta _{2j}$, $j=1,\ldots ,d$, then the model is the same, because only the orientation of the latent variable has been reversed. For simplicity, we report these correlation parameters as being positive for stronger dependence. To make it easier to compare different models, we convert the Gumbel/s.Gumbel and BVN/$t_\nu$ copula parameters to Kendall’s $\tau$’s via the relation in (10) and (11), respectively.

The bigger differences between the factor models with the selected copulas and the factor models with BVN copulas are seen in the estimated parameters or loadings ($\hat{\tau }$s converted to BVN copula parameters $\hat{\theta }_{1j}$ and $\hat{\theta }_{2j}$ with the inverse of the relation in (11) and then to loadings with the relations in Sect. 3.1) for the first and second factor. These are the estimated parameters at tree 1 and tree 2 of the 3-truncated vine which along with the copula choice form the tail asymmetries among the items. The estimates of the factor models with BVN copulas are biased as BVN copulas have zero tail dependence. At the residual dependence part of the model which is the tree 3 of the 3-truncated vine, the differences are negligible as the tail asymmetries (if any) among the items have already accounted in the lower order trees (factor part of the model).

Interestingly, for the factor models with the selected copulas, the Kendall’s $\tau$’s in the 2-factor copula model are roughly equivalent to the estimates in the first and second factors of the 2-factor tree copula model. Most of the dependence is captured in the first two trees, resulting in weak to medium residual dependencies in the 1-truncated vine copula model, but significantly larger from independence. Interpreting the estimated parameters, the latent variable for maxima is positively associated with all items, while the other latent variable is both positively and negatively associated with some of the items. The residual dependencies reveal that there is stronger association between the 10th and 11th items that are “Blame of self or others" and “Negative trauma-related emotions," respectively. In addition, there is moderate association between items 9 and 11 that are “Negative beliefs" and “Negative trauma-related emotions," respectively. With similar moderate dependence found between items 4 and 9 that are “Negative beliefs" and “Emotional cue reactivity," respectively.

**Table 6:** Estimated copula parameters and their standard errors (SE) in Kendall’s $\tau$ scale for the selected 2-factor and 2-factor tree copula models obtained from the partial selection algorithm for the Post-Traumatic Stress Disorder dataset.

	2-factor copula				2-factor tree copula						
	1st factor		2nd factor		1st factor		2nd factor		1-truncated vine		
	$t_2$		Gumbel		$t_2$		Gumbel		$t_5$		
Items	$\hat{\tau }$	SE	$\hat{\tau }$	SE	$\hat{\tau }$	SE	$\hat{\tau }$	SE	$\mathcal {E}$	$\hat{\tau }$	SE
1	$-$0.16 (0.17)	0.06	0.49 (0.50)	0.04	$-$0.17 (0.48)	0.06	0.50 (0.28)	0.04	1, 18	$-$0.18 ($-$0.20)	0.06
2	$-$0.11 (0.18)	0.06	0.49 (0.48)	0.04	$-$0.08 (0.42)	0.06	0.45 (0.29)	0.04	18, 17	0.22 (0.22)	0.06
3	$-$0.14 (0.19)	0.06	0.54 (0.55)	0.04	$-$0.12 (0.49)	0.06	0.52 (0.31)	0.04	18, 14	$-$0.20 ($-$0.13)	0.07
4	$-$0.32 (0.01)	0.06	0.56 (0.64)	0.05	$-$0.34 (0.67)	0.06	0.57 (0.05)	0.05	18, 10	$-$0.10 ($-$0.13)	0.06
5	$-$0.21 (0.11)	0.06	0.55 (0.58)	0.04	$-$0.21 (0.54)	0.06	0.56 (0.23)	0.04	10, 11	0.36 (0.40)	0.05
6	$-$0.13 (0.05)	0.06	0.28 (0.29)	0.05	$-$0.13 (0.27)	0.06	0.26 (0.07)	0.05	11, 9	0.29 (0.30)	0.06
7	$-$0.11 (0.14)	0.06	0.40 (0.39)	0.04	$-$0.09 (0.34)	0.06	0.39 (0.23)	0.04	9, 2	$-$0.18 ($-$0.15)	0.06
8	0.03 (0.16)	0.06	0.21 (0.16)	0.05	0.04 (0.11)	0.06	0.19 (0.19)	0.05	2, 3	0.26 (0.27)	0.06
9	0.17 (0.37)	0.06	0.38 (0.17)	0.04	0.24 (0.04)	0.06	0.33 (0.42)	0.04	3, 20	0.05 (0.11)	0.07
10	$-$0.16 (0.12)	0.06	0.34 (0.35)	0.05	$-$0.12 (0.28)	0.06	0.30 (0.16)	0.04	2, 16	0.13 (0.15)	0.06
11	$-$0.09 (0.25)	0.06	0.52 (0.46)	0.04	$-$0.07 (0.35)	0.06	0.48 (0.34)	0.04	16, 15	0.17 (0.20)	0.06
12	0.23 (0.48)	0.06	0.5 (0.26)	0.04	0.28 (0.14)	0.06	0.50 (0.54)	0.04	9, 4	0.29 (0.34)	0.08
13	0.35 (0.63)	0.06	0.55 (0.14)	0.05	0.34 (0.06)	0.05	0.49 (0.56)	0.05	20, 5	0.05 (0.05)	0.07
14	0.37 (0.55)	0.05	0.41 (0.02)	0.05	0.35 ($-$0.02)	0.05	0.36 (0.50)	0.05	14, 13	0.27 (0.24)	0.07
15	0.09 (0.35)	0.06	0.48 (0.31)	0.04	0.11 (0.22)	0.06	0.44 (0.40)	0.04	5, 6	0.12 (0.13)	0.07
16	0.08 (0.28)	0.06	0.31 (0.20)	0.05	0.10 (0.13)	0.06	0.28 (0.30)	0.04	6, 7	0.23 (0.17)	0.06
17	0.04 (0.29)	0.06	0.34 (0.21)	0.04	0.04 (0.15)	0.06	0.33 (0.31)	0.04	7, 19	$-$0.21 ($-$0.19)	0.06
18	0.06 (0.31)	0.06	0.45 (0.29)	0.04	0.12 (0.25)	0.06	0.46 (0.39)	0.04	16, 8	0.12 (0.11)	0.06
19	0.26 (0.47)	0.06	0.45 (0.16)	0.04	0.28 (0.09)	0.06	0.43 (0.49)	0.04	19, 12	0.08 (0.10)	0.07
20	0.11 (0.42)	0.06	0.41 (0.11)	0.04	0.13 (0.20)	0.06	0.40 (0.37)	0.04	–	–	–

In parentheses, we provide the estimated copula parameters in Kendall’s $\tau$ scale for the 2-factor and 2-factor tree copula models with BVN copulas. The former is equivalent to the bidimensional normal ogive model, and the latter uses the bidimensional normal ogive model as the factor part of the model, while the residual dependence part is a discretized MVN distribution.

## Discussion

We have proposed combined factor/truncated vine copula models to capture the residual dependence for item response data. They form conditional dependence of the items given the latent variables and go beyond the factor models where the items are conditionally independent given the latent variables. By combining the factor copula models with an 1-truncated vine copula model, we construct conditional dependence models given very few interpretable latent variables. The combined factor/truncated vine structure has the form of (i) primary dependence being explained by one or two latent variables, and (ii) conditional dependence of item response variables given the latent variables (Joe, 2018). They are especially useful and interpretable when there are a few latent variables that can explain most but not all of the dependence in the item responses.

The flexibility of the factor tree copula models endorses the significance of model selection. In practice, one has to first select the 1-truncated vine tree structure $\mathcal {E}$ and then, suitable bivariate copulas to account for more probability in the one or both joint tails. We tackle these model selection issues by proposing heuristic algorithms to choose a plausible factor tree copula model that can adequately capture the (residual) dependencies among the item responses. We have shown that the proposed models provide a substantial improvement over the 1-factor and 2-factor (tree) copula models with selected (BVN) copulas on the basis of the AIC and Vuong’s statistics. The 1-factor and 2-factor tree copula models with BVN can be viewed as first-order models if models based on other tail dependent copulas are called. After finding some well-fitting models based on an assumption of a discretized MVN, we can convert to a parameterization with correlations in the first tree and partial correlations in subsequent trees and then, extend to a vine copula model by replacing each correlation by a bivariate copula and each partial correlation by a bivariate copula applied to conditional distributions. We consider the 1- and 2-factor tree copula models to be reasonable parsimonious models as most of the dependence is explained via the first few trees in the factor model. This is because that for all the bivariate margins to have upper/lower tail dependence, it only suffices that the bivariate copulas in the first trees (factor part) to have upper/lower tail dependence and is not necessary for the bivariate copulas in the higher trees after the 1-truncated vine to have tail dependence (Joe et al., 2010).

In the proposed models, the conditional independence and residual dependence parts are modeled separately. The residual dependencies are taken into account by a Markov tree without changing anything to the conditional independence model part. This means that we can remain within a well-known and conceptually attractive framework as offered by the factor copula models when applying a factor tree copula model. This will be attractive to practitioners that have a basic and conceptual understanding of factor models, but are less familiar with complicated models that are available to tackle the problem of residual dependence. The main change in the factor copula model is only in the formulation of the joint conditional distribution, while the conditional part of the model, i.e., the unique loading parameters, these are $\hat{\tau }$s converted to BVN copula parameters $\hat{\theta }_{1j}$ and $\hat{\theta }_{2j}$ with the inverse of the relation in (11) and then to loadings with the relations in Sect. 3.1, is left intact.

## Software

R functions for estimation, simulation and model selection of the factor tree copula models are part of the R package **FactorCopula** (Kadhem and Nikoloulopoulos, 2023b).
